# Influence of Stabilization Techniques Used in the Treatment of Low Back Pain on the Level of Kinesiophobia

**DOI:** 10.3390/ijerph18126393

**Published:** 2021-06-13

**Authors:** Przemysław Filipczyk, Karolina Filipczyk, Edward Saulicz

**Affiliations:** 1 Faculty of Health Sciences, Jan Długosz University in Czestochowa, 42-200 Czestochowa, Poland; 2 Department of Psychiatry, Faculty of Medical Sciences in Zabrze, Medical University of Silesia, 42-612 Katowice, Poland; k.filipczyk@o2.pl; 3 Departhment of Kinesiotheraphy and Special Methods in Physiotheraphy, The Physical Education Academy Jerzy Kukuczka in Katowice, 40-065 Katowice, Poland; e.saulicz@awf.katowice.pl

**Keywords:** low back pain, manual therapy, kinesiophobia, fear of pain, Australian method, Neurac

## Abstract

The aim of this study was to try to compare the effectiveness of manual therapy techniques in combination with stabilization techniques: the so-called Australian method and the Neurac method in relation to pain sensations and the level of kinesiophobia. A total of 69 people were examined, divided into three groups of 23 people each. The Visual Analogue Scale was used to assess the antalgic effect, and the Kinesiophobia Causes Scale questionnaire was used to assess the level of kinesiophobia. Patients improved over four weeks, during which they were assessed three times. The evaluation of the desired parameters was also performed over a 24-week period to assess long-term performance. Stabilization techniques are an effective extension of manual therapy techniques in patients with low back pain. People in the groups additionally improved in terms of stabilization techniques, which are characterized by a lower level of kinesiophobia. Its lowest level was found in the group additionally improved with the Neurac method. In the long-term study, the level of kinesiophobia in this group was still maintained at a reduced level. The use of stabilization techniques involving patients in action may significantly affect the level of kinesiophobia, and thus have a much wider effect than just pain reduction.

## 1. Introduction

The main psychological factor influencing the reappearance and persistence of pain is fear [1,2,3]. As early as in the 1980s, Philips [4] wrote that the fear and avoidance of pain sensations resulted in a behaviour pattern that would lead to an exaggerated perception of pain itself. In patients with acute lumbar pain, it has been found that the fear of symptoms is also associated with reduced participation in daily tasks, especially those with different lifting tasks [5]. These people also perceive their disability as a much more serious phenomenon [6,7]. The feeling of fear intensifies the more and more frequent use of sick leave, which in the long run also creates a risk of job loss [8].

In patients with acute pain, which at the beginning had an increased level of fear of pain sensations, there are more intense experiences related to the self-assessment of their disability [9]. In these people, the avoidance of activities that may intensify the pain sensation is even more pronounced. This is explained, among others, by the Fear Avoidance Model (FAM) [10]. The main assumption of this model is the fear of pain. Fear and avoidance of pain are an important feature of the development of chronic pain in many patients with musculoskeletal disorders. This model suggests that high psychological anxiety will be associated with poor clinical outcomes, potentially resulting in depressive symptoms, increased pain intensity, greater physical disturbance and the development of the further disability of the patient [11]. On the other hand, the chronic condition will have a negative impact on the quality of life [12]. More fear being related to pain may increase the susceptibility to the formation of new pain episodes. Even in currently healthy people, the risk of further ailments increases, which leads to the chronicity of the phenomenon [13]. It has been proven that people suffering from chronic lower back pain avoid risky situations, and if they decide to engage in any activity, it is performed with a very high level of caution, which directly affects the quality of a given motor task [14]. Such conduct will affect the dysfunction of the lumbar muscles, which may potentially lead to even greater limitations in physical activity and an increase in pain sensations [15].

These psychological aspects related to fear are a condition known as kinesiophobia. It is defined as an excessive, irrational and weakening fear of movement and physical activity, resulting in increased sensitivity to repeated, painful damage [16]. The patient can sometimes more or less consciously capture the phenomenon of kinesiophobia in themselves by increasing attention directed at themselves. It is worth mentioning that patients are very often accompanied by “catastrophic thinking”, the greater the pain stimulus caused [17]. It is worth mentioning here that the greater the catastrophic thinking is, the greater the pain stimulus that triggered this thinking will be. Further increase in this phenomenon will also be related to the repetition of the pain stimulus, e.g., during the next painful procedure [17]. The solution to these spirals of abnormalities may be to slowly activate the patients to break the described closed circles that drive the negative phenomenon. The gradual progression of exercise programs is nothing else than the gradual exposure to a fear-inducing stimulus described by Davey [18]. According to the author, it is this approach that will be the most effective component of the treatment of people suffering from excessive anxiety and phobias. Moderate, and above all, gradual exposure to an exercise stimulus, which is also an anxiety factor, may allow the patient to be sure that the therapy is effective, so that the fear of movement is unjustified. 

The direction which allows for the slow activation of the patient in the rehabilitation process can be an interesting solution, especially if it is combined with methods commonly recognized as very effective in antalgic action, such as elements of manual therapy, the Neurac method or the so-called Australian method [19,20]. 

The aim of the study was to evaluate the effectiveness of the antalgic effect and to assess the impact of stabilization techniques on the phenomenon of kinesiophobia.

## 2. Materials and Methods

### 2.1. Participants

**Figure 1:**
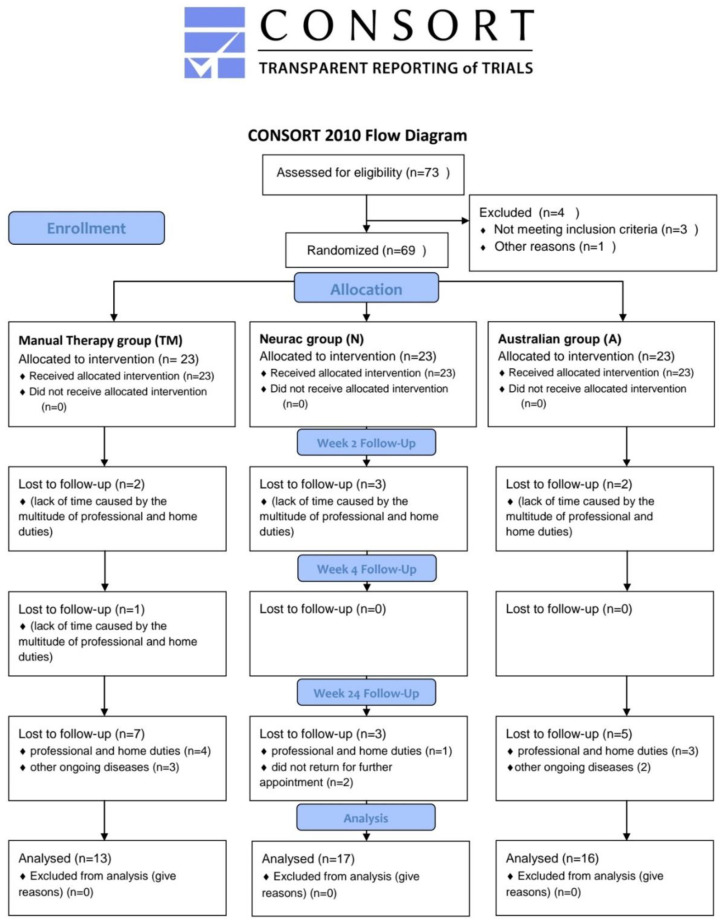
Transparent reporting of trials—CONSORT Flow Diagram.

The participants of the experiment (P) were patients with non-specific lower back pain. Sixty-nine people (37 women and 32 men) participated in the study. The two research groups and the control group each consisted of 23 people at the beginning of the experiment. The total number of people who completed the full cycle of the research experiment program was 46 people (24 women and 22 men), i.e., 66.66% of the respondents who started the experiment. Figure 1 shows the CONSORT Flow Diagram with the transparent reporting of trials at each stage of the experiment. People who did not complete the research process most often explained their deviation from the program by the lack of time caused by the multitude of professional and home duties. At the time of the commencement of the improvement process, the research groups were characterized by a similar level of pain intensity. The analysis of variance (Wilkas test) for the pain scale (VAS) in individual research groups showed that the mean values of the pain scales were not differentiated between the groups (*p* = 0.135). The participants were divided into 3 groups: manual therapy group (TM, n = 23), Neurac group (N, n = 23), and Australian group (A, n = 23). 

Patients were selected for research groups on the basis of systematic random selection. This means that the group improved only with manual therapy techniques consisted of participants who were the first to apply for the study (No. 1 of the respondent), and then every third person participating in the research. Subsequently, it was the persons who reported for the tests numbered 4, 7, 10, etc. A similar random interval concerned the second group-treated with manual therapy techniques and by the Neurac method. In this case, the people who signed up for the group were assigned to group tests of numbers 2, 5, 8., etc. For the third group—treated with techniques of manual therapy and the so-called Australian method—people from numbers 3, 6, 9 were targeted., etc. When applying for the study, 4 people did not meet the criteria for inclusion in the study (3 people had comorbid mental disorders, and one person dropped out). This interval continued until we obtained 23 individuals in each personal research group, giving a total of 69 in the experimental group of patients complaining of non-specific pain ailments.

### 2.2. Ethical Statement

All research procedures were carried out in accordance with the medical experiment design, which received a positive opinion from Resolution No. 6/2013 of 25 April 2013., by University Bioethics Committee for Scientific Research at the Academy of Physical Education “Jerzy Kukuczka” in Katowice.

### 2.3. Study Design

Study design was set as a randomized controlled trial.

### 2.4. Procedure

A diagram of the flow of progress through the study phases in all groups such as enrollment, assignment, intervention, observation, and data analysis is shown in Figure 1.

### 2.5. Intervention

The improvement of the experiment participants was carried out for a period of 4 weeks. During this period, 10 therapeutic meetings were held. They were held on average every 3 days. At that time, there were 4 measurement meetings: before (week 0), during (week 2) and after the rehabilitation period (week 4), and one further study after 6 months (week 24) from the end of therapy.

#### 2.5.1. Manual Therapy Techniques (TM)

The techniques were used in all 3 groups. Therapeutic techniques were used: passive mobilization of intervertebral joints, active mobilization with the participation of the patient, high velocity low amplitude (HVLA) manipulations, traction of the entire lumbar and individual segments, and transverse massage of the paraspinal muscles. The duration of a single therapeutic visit was approximately 15 min. The work with the patient was as follows. After the examination and study of the problem, a trial therapy consisting of mobilization was performed. After the end of the first series, a test was carried out to check what effect the applied technique had on the pain symptoms. If it was possible to reduce them, this technique was used in five series: 10 mobilizations plus a break. The series were repeated five times, and then another test was carried out to see how much pain was reduced in the subject. At the end of the visit, the patient was shown basic exercises that they could safely and independently perform at home [21,22]. These were, among others, hip raises while lying on the back, hip raises combined with taking one leg off the ground, or a relieving position while lying on the back with legs placed on the ball.

**Figure 2:**
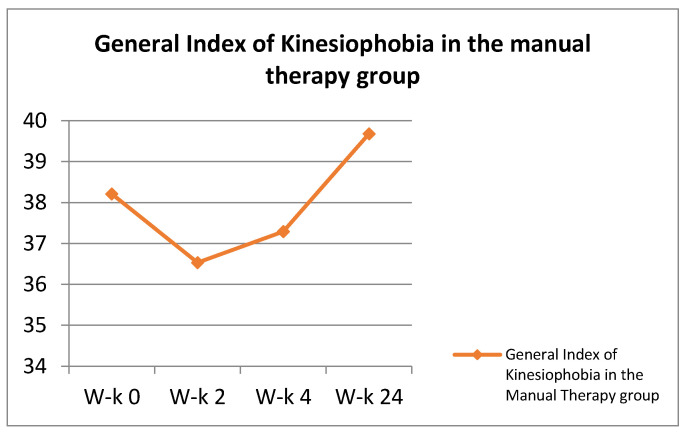
General Index of Kinesiophobia in the manual therapy group.

**Figure 3:**
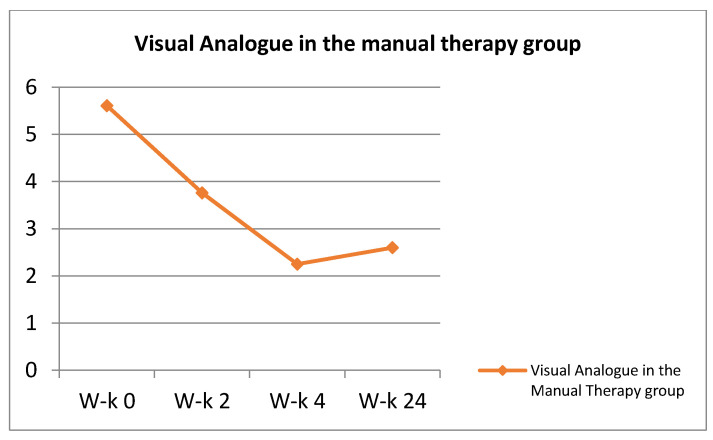
Visual Analogue in the manual therapy group.

In the remaining groups (Neurac method and the so-called Australian method), manual therapy was combined with stabilization training. The single visit time in these groups was increased to approximately 30 min. Manual therapy techniques took 15 min (as in the control group). The remaining time was devoted to stabilization techniques. The combination of manual therapy methods with stabilization methods took place starting with the first visit of the patient. The results regarding the General Index of Kinesiophobia and the VAS scale are presented in the Figure 2 and Figure 3.

#### 2.5.2. Neurac Method (N)

**Figure 4:**
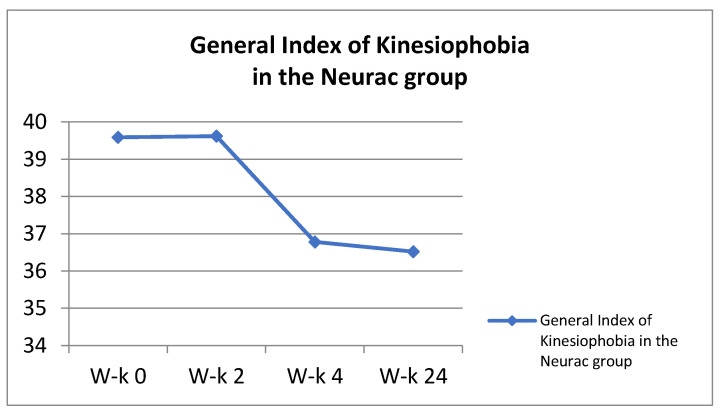
General Index of Kinesiophobia in the Neurac group.

**Figure 5:**
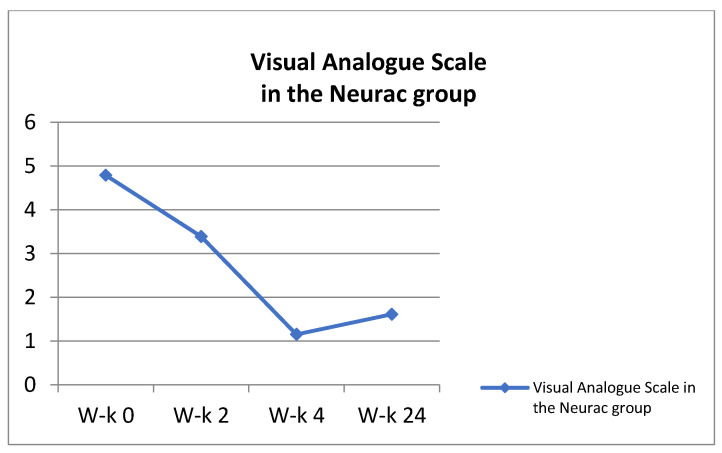
Visual Analogue in the Neurac group.

In the group treated by the Neurac (N) method, four key elements of this method were used, namely exercises using the patient’s own weight, working in suspension; the use of vibrations on selected parts of the body; gradual increase in the load by increasing the workload; and avoiding provocation or increasing pain during exercise. At the beginning of the improvement, relieving elements were used in the form of, for example, elastic lines, which enabled the exercise to be performed. At this stage, the greatest attention was paid to learning the cooperation of various antagonistic muscle groups, shaping the sense of position and joint kinaesthesia, stimulation of neuromuscular activity and exercises in functional movement patterns. The therapy took into account isometric muscle tension, which was maintained as long as the exercise was correctly performed and the patient did not report any pain sensations. Four basic exercises for the core muscles were used, which were carried out using standard principles of exercise progression, allowing for the adjustment of difficulties and loads in each of them. These included: change in the arm length of the acting force; changes in the position of the patient’s body in relation to the suspension point; changing the height of the slings or lines; changing the level of instability; performing additional movements; and the application of additional weights. While working with the patient, the following operating strategy was used: the exercises were performed from 3 to 6 repetitions in 2–4 series. The intervals between the series were approximately 30–60 s. New exercises were started at such a level of difficulty that the exercising person could correctly perform the motor task and without experiencing pain. The level of difficulty was passed to the next level when the patient was able to perform the assumed number of repetitions in the last series at a lower level, while maintaining the appropriate quality of movement [23]. The aforementioned 4 basic elements were carried out through unloading exercises performed in closed kinematic chains, taking into account the patient’s body weight and unstable ground through the use of slings. In addition, in the exercises, mechanical disturbances consisting of shaking the lines and slings were used to further enhance the instability effect. The greatest attention was devoted to learning the cooperation of various antagonistic muscle groups, shaping the sense of position and joint kinaesthesia, the stimulation of neuromuscular activity and exercises in functional movement patterns [24]. The therapy took into account isometric muscle tension, which was maintained as long as the exercise was correctly performed and the patient did not report any pain sensations. Increasing the exercise time in which the subject was able to hold the position increased with the repetitions, and at the same time, the time in which the position was held increased. This progression was a way of moving to further stages of work according to the Neurac method, thus taking into account the so-called the progression ladder [25]. The results regarding the General Index of Kinesiophobia and the VAS scale are presented in the Figure 4 and Figure 5.

#### 2.5.3. The So-Called Australian Method (A)

It is mainly based on the research of C. Richardson, G. Jull, P. Hodges and J. Hides conducted at the University of Queensland in Brisbane, proving that the stabilization mechanism of the lumbar spine and pelvis is disturbed in people with pain and overload in the lower spine [26]. Therapy with the use of the so-called Australian method was conducted based on the methodology developed by Peter B O’Sullivan [27]. Rehabilitation in group A began with learning to tighten the deep abdominal muscles in the supine position with bent lower limbs, in the forward lying position and in a supported kneeling position. The way to repeat the exercise and build the ability to self-check whether the tension is correct was by palpation. The patient was instructed to gently and slowly tense the abdominal walls so that their lower part was pulled in and a slight tension appeared under the fingers. If a person showed a problem with this task, additional variants of learning to activate the transverse abdominal muscle were used, e.g., sono-feedback. The described method of therapy was its first stage, lasting about 2–3 meetings, i.e., until the patients mastered the technique of the exercises. The stabilization training in group A consisted of 3 stages. In the first (described above), local segmental control was launched by activating and training the local muscle system.

In the second stage, participants learned segmental control in closed kinematic chains. The aim of this stage was to further develop the segmental control of individual joints by activating and training local muscles in conjunction with the muscles of the anti-gravity system. Biofeedback was used in all exercises thanks to the possibilities offered by a stabilizer device equipped with a blood pressure meter [28]. An exemplary exercise at this stage, performed in the supination position, was to maintain a constant pressure in the stabilizer while retracting the abdomen for approximately 10–15 s. The pressure in the inflated device was 40 mmHg. The device was placed under the lumbar spine (approximately at the height from S2 to L1 [29]. Increasing pressure means the excessive activity of the rectus abdominis and external oblique muscles; similarly, reducing the pressure in the “stabilizer” will mean a weakening of the activity of the deep muscles. There were also exercises in the pronation position. In this case, the exercise cushion was placed so that its lower line was aligned with the line formed by the upper front hip spikes. In the supine position, pulling the abdomen should reduce the pressure in the “stabilizer” (at the initial value of 70 mmHg) by 6–10 mmHg and stay at this level for about 10–15 s. At this stage, the subjects were taught to control the deep muscles while performing closed-chain movements. The suggested activity consisted, for example, in bending the knee in a supination position [30].

**Figure 6:**
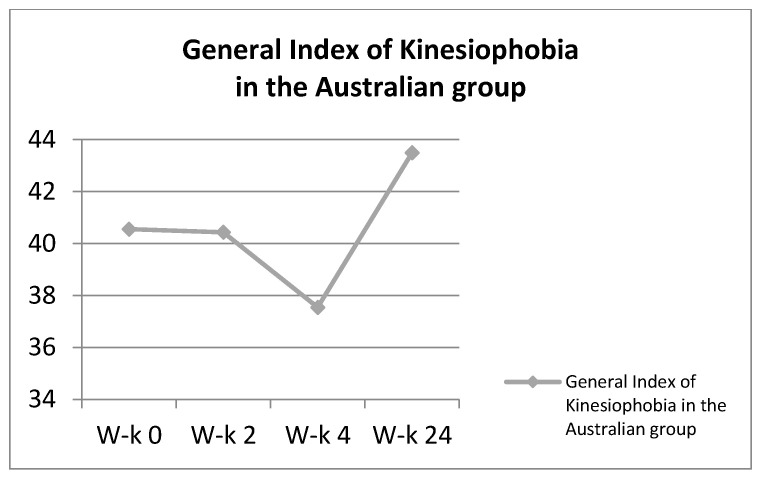
General Index of Kinesiophobia in the Australian group.

**Figure 7:**
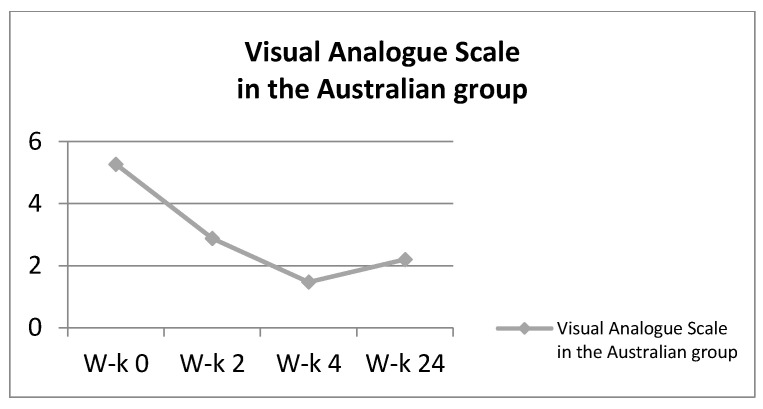
Visual Analogue in the Australian group.

In the third stage of improvement, the subjects learned through exercises how to correctly segment control in open kinematic chains. At this stage, the described exercises were performed by the patients under the additional load of lifting the straightened lower limb. The re-education of the tension of the deep muscles of the lower torso in the standing and sitting position was a necessary development of stabilization exercises. At each stage of work with the patient, the therapist observed the work of the subject in terms of correctness and conveyed comments when performing the task incorrectly. During the exercises, a similar work pattern was maintained at all stages. The patient performed approximately 8–10 repetitions of each exercise. The break between them was about 10 s, and the break between other exercises was selected individually and was usually about 2 min. The results regarding the General Index of Kinesiophobia and the VAS scale are presented in the Figure 6 and Figure 7.

### 2.6. Outcome Measures

To determine the level of kinesiophobia, we used a questionnaire that allowed to assess the degree of kinesiophobia in patients participating in the experiment: the Kinesiophobia Causes Scale (KCS) questionnaire [31]. This questionnaire is used to determine the barriers to physical activity in two domains: biological and psychological. Thanks to the answers given (on a scale of 0–100), presented as a percentage, it is possible to determine the level of the intensity of barriers to physical activity. The biological domain (DB) is the average of the factors-parameters: morphological, stimulation demand, energy resources and the strength of biological drives. The psychological domain (DP) is the average of points from factors such as: self-acceptance, motor skills, well-being and social influence. The overall kinesiophobia index (OWK) is half the sum of the biological and psychological domain points. The total score of the questionnaire ranges from 0 to 100 and can be expressed as a percentage of kinesiophobic behaviour. The higher the KSC index in the subjects, the higher the level of kinesiophobia [32].

To determine the level of pain intensity. The effectiveness of the antalgic effect was assessed using one of the most accurate pain scales—the Visual Analogue Scale (VAS). The scale is a 10 cm line. The patient marks a point on it which indicates the intensity of pain from 0—no pain at all; to 10—the strongest pain imaginable. [33].

### 2.7. Statistical Analysis

All the collected numerical data were subjected to statistical analysis in STATISTICA version 12.0 by StatSoft. The basic descriptive statistics procedures were performed, in which mean values (x), standard deviation (sd) as well as minimum (min) and maximum (max) values were calculated. ANOVA was used to compare the clinical status between the groups analysed on the basis of the questionnaires and the pain scale. Levene’s test was also performed as a control. If it showed significant differences in the homogeneity of variance; then, the Kruskall–Wallis test was performed instead of ANOVA. In both cases, a post hoc test was performed. The Bonferroni post hoc test was also used to compare the intergroup effects, inter-study effects and interactions between groups.

## 3. Results

Characteristics of the respondents from particular groups in terms of basic biometric data are presented in Table 1.

The analysis of variance for repeated measurements of the VAS showed that the mean values of the obtained results were not differentiated between the groups (*p* = 0.12). The interaction effect is not statistically significant (*p* = 0.78), so the effectiveness of therapy in individual research groups was similar.

**Figure 8:**
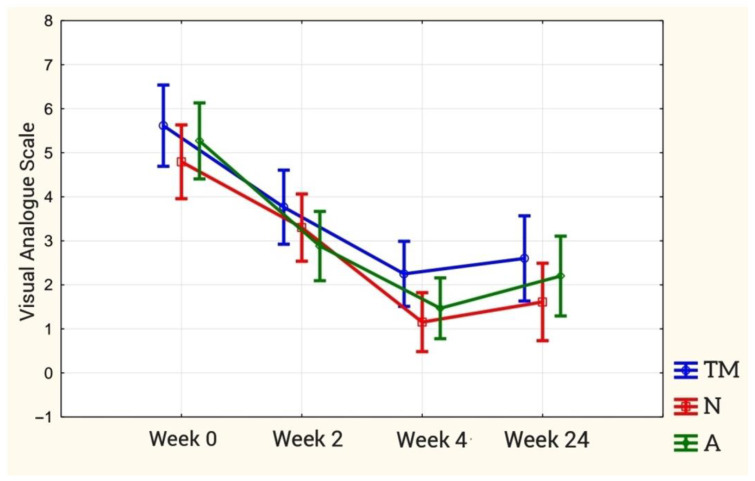
The course of variability of pain intensity according to the VAS scale in individual stages of therapy in relation to research groups.

The therapeutic effect of changing the pain sensation at particular stages of the study is statistically significant (*p* < 0.00). Post hoc analysis showed statistically significant differences in the baseline (week 0) and final study (week 4), and the baseline (week 0) and late study (week 24) in each study group (*p* < 0.001 for each group). In the initial and intermediate study, there were differences between the TM and A groups (*p* = 0.015). However, these were not present in the N group (*p* = 0.055). The results are presented in Figure 8.

There are no statistically significant differences between the final examination and the follow-up examination in the TM and A groups. However, in the N group, these differences are statistically significant.

**Figure 9:**
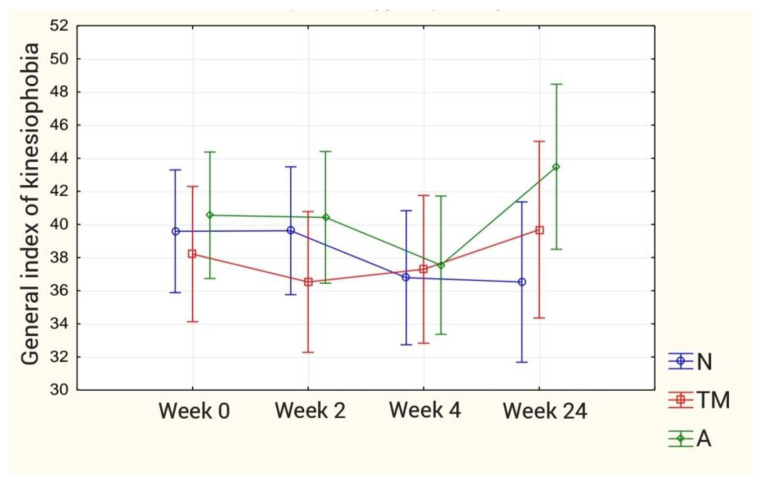
Mean values of the General Index of Kinesiophobia in individual groups.

For the Kinesiophobia Causes Scale (KCS): The analysis of variance with the ANOVA test for all components of the questionnaire showed a statistically significant difference between the individual measurements (biological dominant) and in the group-study interaction (psychological dominant). Further analysis with the Bonferroni post hoc test showed no statistically significant differences from the biological dominant and the overall kinesiophobia index for both intergroup effects, inter-study effects and group-study interaction. On the other hand, for the psychological dominant, the post hoc test revealed a statistically significant intergroup difference regarding distant measurements (*p* = 0.044) between group N and group A. The results are presented in Figure 9. The results concerning the kinesiophobia index are presented in Table 2.

## 4. Discussion

The results of the presented study confirm the hypothesis that the therapy that combines various stabilization techniques with manual therapy techniques is an effective form of work with the patient.

Physical activity affects our body in many ways. Many studies show that being active can alleviate symptoms of depression, reduce psychotropic medications, and provide additional therapeutic benefits beyond psychotherapy.

There are many studies showing that adapted physical activity, mainly through specific exercise, is very beneficial for the patient in a biomedical sense. It improves strength, supports the healing process, and even the fusion of bones, ligaments or muscles [34].

In the context of this work, however, exercise is primarily associated with psychological benefits, in contrast to bed rest and other forms of immobility, which are simply disastrous for structures such as discs, muscles, joints, bones and ligaments [35]. An interesting study similar to the presented study compares the therapeutic benefits of stabilization exercises in patients with various levels of anxiety measured before being active. They showed that in the case of people in which higher levels of kinesiophobia have been detected, a regular exercise program was more effective than standard medical care [36]. In the experiment, the subjects used various therapeutic methods. However, only in the N method were they led in a seemingly greater load, such as stabilization exercises in suspension. Perhaps it was this fact which contributed to the better results presented by the subjects from the N group in the distant study. The better effect in the N group may be due to the fact that in this method of rehabilitation, the patient is gradually “exposed” to greater effort, and the gradual progression of the difficulty level of the exercises helps to get rid of the fear of this activity. At the same time, this approach seems to perfectly fit into the assumptions of cognitive behavioural techniques, which use situations that are “dangerous” or “threatening” in the opinion of the patient, while explaining what exactly the fear avoidance model is. Then, adapted exercise tasks are introduced based on the hierarchy of fear-inducing situations [37]. In the Neurac method, however, we refer to this hierarchy as the “progression ladder”.

Using the patient’s individual symptoms, beliefs and methods of avoidance, it is possible to clearly explain the operation of the vicious circle that maintains the problem of pain for a long time. In the other methods used in the experiment, the subjects were not so exposed to effort. They did not risk confrontation with a greater load, which is that of performing stabilization exercises while suspended, but performed tasks in stable positions, such as lying or sitting. One could consider whether the so-called Australian method supplemented with manual therapy techniques or the lack of any additional method and using only manual therapy measures would yield similar results. Considering only the fear of being active, this may be the case.

Nevertheless, it should be remembered that working with a patient suffering from LBP is a multi-factor task in which pain plays the leading role. Pain, as the most pronounced sensation, is often the only one that forces the patient to seek help. Additionally, for the pain factor itself, each of the methods used in the experiment works effectively, reducing it to a significant extent. Moreover, in each of the research groups, the effectiveness of the therapy in terms of pain relief was similar, even in a long-term study. Kinesiophobia can also develop the so-called “disuse syndrome”. In other words, the fear of pain will be responsible for the deterioration of physical condition and the formation of abnormal movement patterns, often seen in patients with chronic pain. Of course, this condition will not only be influenced by the fear of being active, but also by avoiding activity, and thus weakening physical strength [38].

The fear of being active further increases the patient’s excessive vigilance, the vigilance focused on avoiding the source of the threat, but also the sources that may cause (according to the patient) a similar effect. Excessive vigilance occurs when a person is constantly engaged in searching for potential sources of danger in the surrounding environment. Of course, this condition will be associated with a decrease in the willingness to expose oneself during any activity. Additionally, the person will only show selective participation in activities which, according to them, may be associated with threat. Both the mere avoidance of participation in certain activities and the described excessive vigilance reduce anxiety, but only in the short term. In the long run, these may be counterproductive [39].

Determining the level of kinesiophobia in patients before starting the rehabilitation process may be of fundamental importance for a multi-disciplinary view of the problem of improving non-specific lower back pain. People with higher levels of kinesiophobia respond well to coping strategies for everyday situations. A simple and clear commentary on the way of performing certain activities, but also explaining the phenomenon of kinesiophobia itself, discussing the strategy of not avoiding activity, may turn out to be a milestone in improving LBP patients.

### Limitations

The topic related to the choice of the type of combination therapy, taking into account the goal of not only the reduction in pain, but also the reduction in kinesiophobia in patients with LBP, is still on the verge. Additionally, the issue itself requires a lot of analysis. It should also be remembered that the group of people who have completed the experiment process is relatively small, which does not enable us to confidently draw far-reaching conclusions about kinesiophobia. Nevertheless, this seems to be an interesting topic and worth developing in the context of reducing kinesiophobia in LBP patients.

The baseline characteristics showed significant differences in age and BMI in group N. Although the statistical analysis did not show significant differences in the baseline studies between the groups in the context of the assessment of the VAS scale and the KCS questionnaire, it should be taken into account when developing these studies. The lower BMI index and the age of the respondents could have contributed to the better acceptance of the load during exercise by this group of respondents. Therefore, increasing the number of subjects, and reducing the number of subjects, could prove to be valuable.

## 5. Conclusions

1. The therapy that combines various stabilization techniques with manual therapy techniques is an effective form of work with the patient. This method of therapy gives a long-lasting analgesic effect.

2. The effect of reducing the level of kinesiophobia is visible in each of the research groups (N and A), as well as in the control group (TM). However, it was only in the N group, where the patients were challenged by more demanding exercises compared to other research groups, that the effect lasted the longest and was visible in the distant study.

## Figures and Tables

**Table 1:** Characteristics of the respondents from particular groups in terms of basic biometric data.

Feature	TM	A	N	*p*-Value
**Gender**	13 W (18.84%) 10 M (14.49%)	13 W (18.84%) 10 M (14.49%)	12 W (17.39%) 10 M (15.94%)	0.943 ^1^
**Age**	50.4 ± 10.3 32–64	45.4 ± 9.11 27–64	37.2 ± 10.4 20–60	<0.001 * ^2^
**Body height**	169.7 ± 10.0 152–187	171.4 ± 7.1 159–187	173.9 ± 9.7 154–190	0.288 ^2^
**Body weight**	77.3 ± 14.9 50–106	73 ± 9.3 52–89	70.9 ± 13.7 50–95	0.243 ^2^
**BMI**	26.68 ± 3.8 16.9–32.9	24.9 ± 3.2 19.3–34.8	23.3 ± 3.1 18.8–29.7	0.005 * ^2^

^1^—Pearson Chi-square test; ^2^—ANOVA test; TM—manual therapy group; N—Neurac group; A—Australian group; W—woman; M—Man; * statistically significant result.

**Table 2:** Therapeutic effect in relation to the KCS questionnaire.

Dependent Variables	*TM*				*N*				*A*				ANOVA Test *p*-Value		
	Wk 0	Wk 2	Wk 4	Wk 24	Wk 0	Wk 2	Wk 4	Wk 24	Wk 0	Wk 2	Wk 4	Wk 24	Group	Research	Interaction
**DB**	32.43 ± 2.22	31.36 ± 2.76	29.19 ± 2.46	32.78 ± 3.16	34.82 ± 2.02	32.83 ± 2.51	29.82 ± 2.23	31.49 ± 2.87	32.25 ± 2.08	32.40 ± 2.59	27.90 ± 2.30	32.84 ± 2.96	0.936	0.035 *	0.926
**DP**	44.00 ± 2.52	41.70 ± 2.51	45.39 ± 2.64	46.59 ± 3.18	44.35 ± 2.28	46.42 ± 2.28	43.74 ± 2.40	41.55 ± 2.89	48.86 ± 2.35	48.47 ± 2.35	47.18 ± 2.47	54.13 ± 2.97	0.134	**0.366**	0.006 *
**OWK**	**38.21 ± 2.03**	**36.53 ± 2.11**	**37.29 ± 2.21**	**39.68 ± 2.65**	**39.59 ± 1.84**	**39.62 ± 1.92**	**36.78 ± 2.01**	**36.52 ± 2.40**	**40.55 ± 1.90**	**40.43 ± 1.97**	**37.54 ± 2.07**	**43.49 ± 2.47**	**0.510**	**0.120**	**0.153**

DB—biological dominant; DP—psychological dominant; OWK—General Index of Kinesiophobia; Wk—Week 0–24; * statistically significant result.

## Data Availability

The data that support the findings of this study are available from the corresponding author, upon reasonable request.
